# The application of metagenomics in the detection of arboviruses in mosquitoes (Diptera: Culicidade): a systematic review

**DOI:** 10.1590/S1678-9946202567046

**Published:** 2025-07-07

**Authors:** Everson dos Santos David, Shirley Vasconcelos Komninakis, Erique da Costa Fonseca, Anne Caroline da Silva Soledade, Karen Carmo dos Santos, Raimundo Nonato Picanço Souto

**Affiliations:** 1 Universidade Federal do Amapá, Departamento de Ciências Biológicas e da Saúde, Laboratório de Arthropoda, Macapá, Amapá, Brazil; 2 Universidade Federal do Amapá, Programa de Pós-Graduação em Biodiversidade e Biotecnologia na Amazônia Legal (Rede Bionorte), Macapá, Amapá, Brazil; 3 Universidade Federal de São Paulo, Departamento de Medicina, Laboratório de Retrovirologia, São Paulo, São Paulo, Brazil

**Keywords:** Viral metagenomics, Mosquitoes, Arboviruses, Sequencing, PRISMA

## Abstract

Advances in deforestation and climate change directly cause changes in habits and the distribution of Culicidae across the globe, especially mosquitoes of medical importance and the main vectors of arboviruses. The viral metagenomics technique can be an important tool in characterizing viral diversity in mosquitoes. Thus, this study aimed to identify evidence of the effectiveness of the viral metagenomics technique in detecting arboviruses in mosquitoes. This is a systematic review based on the Preferred Reporting Items for Systematic Reviews and Meta-Analyses (PRISMA) 2020 protocol. The research was carried out using five electronic databases: LILACS, PubMed, SciELO, Scopus, and Web of Science, and included studies published in health and interdisciplinary fields, as well as complementary research on Google Scholar. Studies that used the viral metagenomics approach for the genomic evaluation of arboviruses found in mosquito samples were included; the results demonstrated the presence of viral diversity and the identification of the genome of probable pathogenic viruses. The protocol was registered on the International Prospective Register of Systematic Reviews (PROSPERO) platform under the number CRD42024484713. Thus, 249 studies were identified via searches on electronic databases. According to the inclusion/exclusion criteria, only 23 studies met the objectives for the systematic review. In all studies, genomic sequencing was applied to detect viruses, mainly those related to insect-specific viruses (ISV) and arboviruses known to infect humans and animals, belonging to various viral families. Despite the challenges reagrdingthe absence of reference sequences in genomic databases, the effectiveness of the metagenomics technique in characterizing the mosquito virome is clear from the studies, which broadens the understanding of viral diversity.

## INTRODUCTION

The spread of arboviruses around the world is favored by the high diversity of vectors, mainly mosquito species from the Culicidae family, due to their ability to transmit pathogens to humans and animals^1^. Environmental, climatic and seasonal changes favor the occurrence and maintenance of transmission cycles of various arboviruses, mainly due to the zoonotic routes that are maintained in enzootic transmission cycles, a predominant factor that highlights the importance of public health^2^.

In this way, it should be noted that arboviruses are widely distributed across the globe and are adapted to remote areas with high and low temperatures^3,4^. There are approximately 600 species of arboviruses, of which some 150 are responsible for pathogens in humans and animals, considered accidental hosts. The risk of infection is directly associated with exposure to environments where the viruses circulate^4,5^.

Among the most relevant arboviruses are the dengue virus (DENV), the chikungunya virus (CHIKV), and the Zika virus (ZIKV), transmitted mainly by the *Aedes aegypti* species^6^; the yellow fever virus (YFV) transmitted in the wild cycle by mosquitoes of the *Haemagogus* and *Sabethes* genera, and in the urban cycle by the *Aedes aegypti*; the Mayaro virus (MAYV), transmitted by the mosquito *Haemagogus janthinomys* ^7^; the Oropouche virus (OROV) has already been found naturally infecting *Coquillettidia venezuelensis* species^8^; the Saint Louis encephalitis virus (SLEV) transmitted by mosquitoes of the *Culex* genus^9^; and the Rocio virus (ROCV), isolated from *Psorophora ferox* species^10^.

In this sense, the viral metagenomics technique has proven to be efficient in detecting viruses when applied to different samples, such as plasma^11^, feces^12^, and in the various extreme habitats^13^. If enabled, its application in mosquito samples can be effective in identifying arboviruses, due to the sensitivity of the technique in detecting the viral genomes that coexist in a given sample, contributing directly to the epidemiological surveillance of the main arboviruses^14^.

This systematic review aims to search evidence of the effectiveness of the viral metagenomics technique in detecting arboviruses in mosquitoes, enabling studies of the viral genetic variability of samples from different areas and the identification of emerging and re-emerging viruses that can cause epidemics and even future pandemics.

## MATERIALS AND METHODS

This is a systematic review based on the Preferred Reporting Items for Systematic Reviews and Meta-Analyses (PRISMA) 2020 protocol^15^, adapted according to recent systematic review studies^16-18^. It is registered in the International Prospective Register of Systematic Reviews (PROSPERO) under number CRD42024484713.

### Detection of arboviruses

The research question was formulated using the acronym PICo as an auxiliary tool^19^. PICo is made up of three components, namely Population or Problem, Interest and Context. Three aspects were addressed in this review based on these three components: detection of arboviruses in mosquitoes (problem), analysis of the effectiveness of the metagenomics technique (interest) and detection of arboviruses using the technique (context). Thus, the following research question was elaborated: what is the evidence of the effectiveness of the viral metagenomics technique in detecting arboviruses in mosquitoes?

The research took place from April to July 2024, and was later updated in April 2025, using five electronic databases: LILACS, PubMed, SciELO, Scopus and Web of Science. It included studies published in health and interdisciplinary fields in English, Spanish and Portuguese, with no restrictions on the date of publication. To define the search terms, the selection of Health Sciences Descriptors (DeCS) and Medical Subject Headings (MeSH) in English was used for expansion via title and abstract.

**Figure 1:**
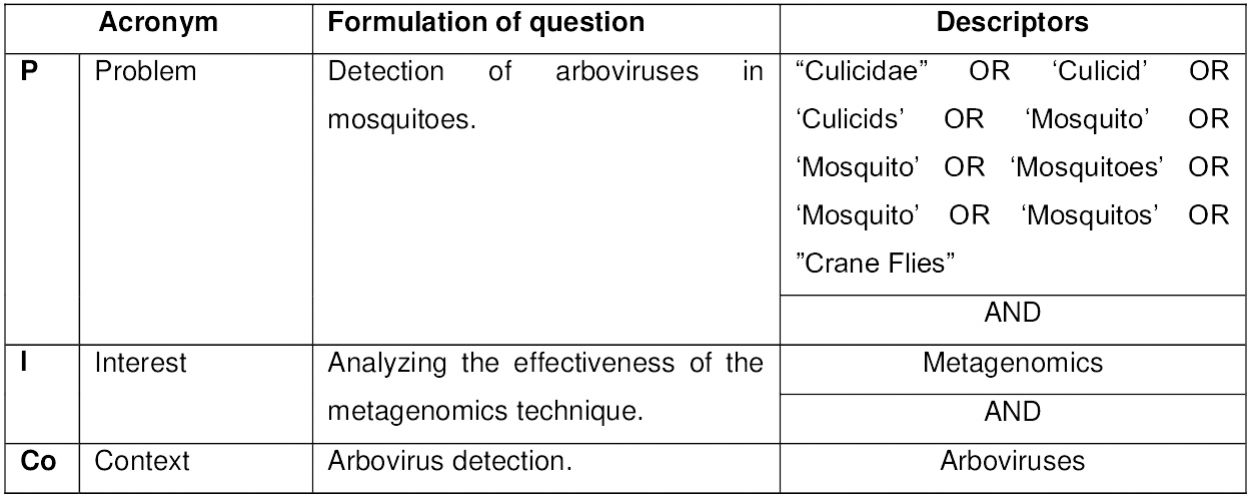
Formulation of the question based on the PICo acronym and the search strategy used for the systematic review.

The search strategy was based on the descriptors mentioned in Figure 1. A manual search was also carried out on the references of the selected articles, as well as on the Google Scholar, following the search strategy presented in Figure 1, to identify relevant studies that were not indexed.

### Eligibility criteria

Studies aimed at detecting arboviruses in mosquitoes were considered, specifically those belonging to the Culicidae family, especially the ones of genera that are considered to be of medical importance, such as *Anopheles*, *Culex* and *Aede*s, vectors of pathogens in humans. Studies that used the viral metagenomics approach for the genomic evaluation of all viruses existing in mosquito samples were included. The results demonstrated the presence of viral diversity and the identification of the genome of probable pathogenic viruses.

Primary studies with an experimental design were included—whether prospective, retrospective or cross-sectional—carried out in any region of the world. Studies related to study objects such as ticks, blood samples or other insects analyzed by metagenomics were excluded.

### Data extraction and evidence synthesis

The files resulting from the database search were transferred to the Mendeley reference manager, version 1.18, in order to remove duplicates. The Rayyan QCRI platform was then used to select articles. The first stage consisted of a title and abstract analysis conducted independently by two researchers (ESD and ECF). Disagreements were resolved independently by a third researcher (ACSS). After this stage, the full text was read independently by two authors (ESD and ECF). In addition, the reference list of the included studies was reviewed for potentially eligible studies not identified in the database search.

Data extraction was performed independently by two reviewers (ESD and ECF) using an extraction form in Microsoft Excel 2016 (Microsoft, Redmond, WA, USA). Discrepancies were resolved by consensus with a third researcher (ACSS). The data extraction form for the eligible studies included the following topics: genera of the Culicidae family, species, year of study, study objective, study area, habitat characteristics, country, mosquito distribution, number of pools, arboviruses identified in the samples, and the sequencer used in the research.

### Quality assessment

In order to assess the risk of bias, the Systematic Review Center for Laboratory Animal Experimentation (SYRCLE) tool for animal studies was used^20^. This tool contains the following evaluation categories: selection bias, performance bias, detection bias, attrition bias, reporting bias and other sources of bias. Ten questions were applied to the articles included in the systematic review, the answers to which can be “YES,” indicating a low risk of bias, “NO,” indicating a high risk of bias, and “UNCERTAIN,” indicating an uncertain risk of bias. It is not recommended to calculate the sum score of each individual study using this tool.

## RESULTS

### Characteristics of the included studies

**Figure 2:**
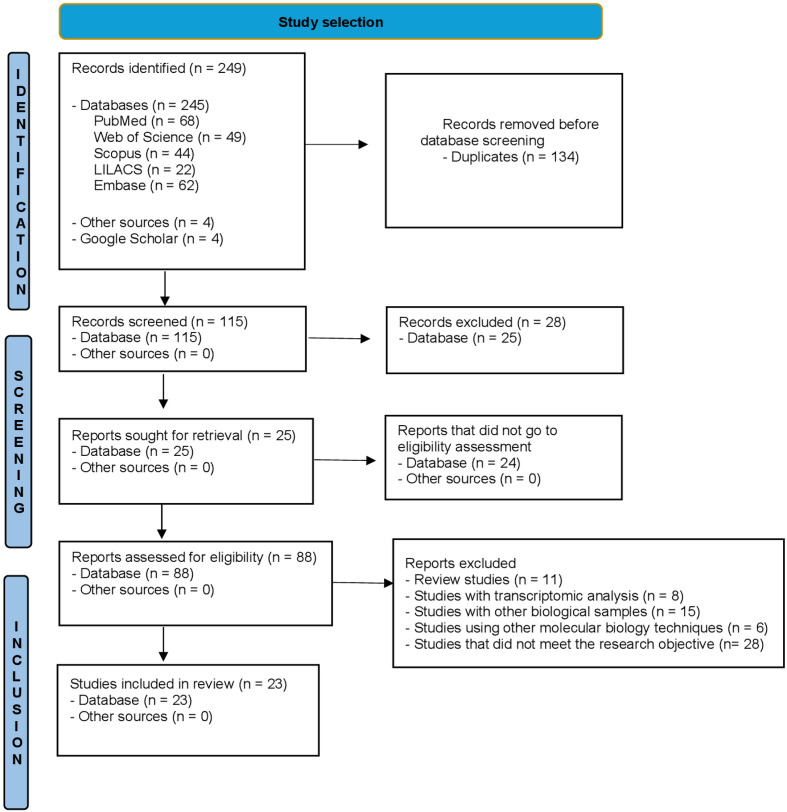
PRISMA 2020 flow diagram.

Figure 2 shows that 249 studies published in the electronic databases were identified following the criteria of the PRISMA protocol^15^. After initially reading titles and abstracts, 88 studies were screened for full text. According to the inclusion/exclusion criteria, only 23 studies were eligible for this systematic review.

The main characteristics of the 23 studies included in this systematic review are shown in Table 1. The studies were published between 2016 and 2025, in different parts of the world, spread across five continents, and aimed to detect arboviruses in mosquitoes (Diptera: Culicidae) using the metagenomic approach.

**Table 1:** Main characteristics of the studies included in the systematic review

	Article	Place	Mosquito species	Number of pools/ specimens	Number of mosquitoes	Year of collection
1.	Sanborn *et al*.^30^	Gyeonggi Province, South Korea	*Culex bitaeniorhynchus* *Culex tritaeniorhynchus*	260 / 1–39	78,907	2018
2.	Williams *et al*.^21^	Kimberley Region of Western Australia	*Culex annulirostris*	12 /25	20,556 - 300 were processed.	2017 and 2018
3.	Calle-Tobón *et al*.^6^	Medellin, Colombia	*Aedes aegypti* and *Aedes albopictus*	14 / 15–36	430	2015 and 2019
4.	Reuter *et al*.^32^	Hungary	*Aedes vexans*spp*.* *Anopheles hyrcanus* *Culex* *Ochlerotatus* spp*.*	9	348	2013 and 2014
5.	Öhlund *et al*.^35^	Sweden	*Coquillettidia richardii,* *|Aedes communis,* *Aedes annulipes,* *Aedes cantans,* *Culex pipiens* and *Culex torrentium*.	12 /8–12	953	2017
6.	Kubacki *et al*.^2^	Switzerland	*Aedes albopictus*	15 /5–59	538	2019
7.	He *et al* ^.^3	Shaanxi-Gansu-Ningxia, China	*Culex pipiens ,* *Culex tritaeniorhynchus ,* *Anopheles sinensis,* *Aedes* spp.	22	22,959	2019
8.	Thannesberger *et al*.^31^	Barbados	*Aedes aegypti,* *Culex pipiens*	27	3,000	2016
9.	Omuoyo *et al*.^22^	Kenyan Coast	*Aedes aegypti*	826 /20	16,520	2017
10.	Sadeghi *et al*.^40^	California, USA	*Culex* sp*.*	51	12,000	2016
11.	Ribeiro *et al*.^34^	Macapa, Amapa and Santos (SP), Brazil	*Aedes aegypti* and *Culex quinquefasciatus*	191	Not informed	2017
12.	Oguzie *et al*.^39^	Nigeria	*Aedes aegypti,* *Aedes simpsoni,* *Aedes luteocephalus* *Anopheles coustani* and *Aedes albopictus.*	26 / 50	1,300	2017 and 2020
13	Atoni *et al* ^.2^6	Kenya and China	*Culex* spp.	247 / 50	12,350	2014, 2015 and 2017
14	Faizah *et al* ^.2^8	Ishikawa, Tottori and Nagasaki, Japan	*Culex tritaeniorhyncus and* *Culex Pseudovishnui*	13	652	2017
15	Hameed *et al* ^.2^9	Yunnan and Myanmar, China	*Culex tritaeniorhyncus* *Culex quinquefasciatu,* *Anopheles sinensis,* *Anopheles minimus,* *Armigeres subalbatus,* *Armigeres obturbans* and *Aedes aegypti.*	10	4,576	2018
16	Sanborn *et al*.^30^	Republic of Korea	*Aedes vexans nipponii,* *Culex pipiens* and *Culex tritaeniorhynchus*	736	6,368	2016
17	Shahhosseini *et al*.^33^	Benin, Iran, Danube, Romania, and Rio de Janeiro	*Aedes* spp*.* *Aedes aegypti* *Culex* spp*.*	4,173 / 1– 250	261,521	2012 and 2015
18	Mbigha *et al*.^36^	Cameroon	*Aedes albopictus* *Aedes simpsoni* *Aedes africanus* *Aedes aegypti*	54 / 4	398	2020
19	Gómez *et al*.^38^	Colombia	*Aedes aegypti*	40	200	2020 and 2021
20	Guimarães *et al*.^43^	Sao Paulo, Brazil	*Anopheles strodei* *Culex chidesteri* *Culex renatoi* *Culex quinquefasciatus* *Culex (Cux.)* sp. *Culex (Mel.)* sp. *Aedes scapularis*	26 / 20 e 50	886	2020
21	Li *et al*.^45^	Bangladesh	*Anopheles* spp*.*	150	644	2018 and 2019
22	Guimarães *et al*.^46^	Sao Paulo (SP), Brazil	*Anopheles strodei* *Culex chidesteri* *Culex renatoi* *Culex quinquefasciatus* *Culex (Cux.)* sp. *Culex (Mel.)* sp. *Aedes scapularis*	25 / 20 e 50	886	2020
23	De Coninck *et al*.^47^	Belgium	*Aedes japonicus* *Culex pipiens molestus Culex* *pipiens pipiens* *Culex torrentium*	198	198	2019 and 2020

However, the search strategies in the databases enabled the analysis of a range of results from the metagenomic approach, enabling not only the detection of arboviruses, but also an understanding of the diversity of viruses present in mosquitoes. Thus, it was demonstrated that the metagenomic technique has the potential to detect human and animal pathogens without the need for cell culture or the use of animals and insect-specific viruses (ISVs) found exclusively in mosquitoes^21^.

The mosquito species shown in Table 1 refer to species predominance in a given geographical area with an incidence of important and emerging arboviruses, areas chosen based on their endemic importance. All studies aimed at collecting mosquitoes in areas with recorded arbovirus outbreaks.

The objective of the studies was to capture female mosquitoes as they are hematophagous and acquire the nutrients necessary for the viability of reproduction by sucking blood. In this way, they inoculate humans and animals with pathogens, causing diseases^22^.

Mosquitoes are taxonomically classified in the order Diptera, suborder Nematocera and family Culicidae. They have approximately 3,618 described species grouped into two subfamilies: Anophelinae and Culicinae. Thus, the eligible studies targeted these two subfamilies, specifically the species belonging to the *Anopheles*, *Aedes* and *Culex* genera, because these vectors can transmit pathogens to humans and vertebrate animals, and are more efficient in transmitting pathogens^23,24^.

Of the 23 eligible studies, 17 obtained results on the diversity of *Aedes* mosquito viruses, 10 applied the metagenomics method to the *Aedes aegypti* species and two to *Aedes albopictus.* Both species are considered cosmopolitan due to their occurrence on all continents and their capacity to thrive in the same types of breeding grounds^25^.

Species of the genus Culex were predominant in 16 studies, as they are an efficient vector for transmitting the West Nile virus, the Rift Valley fever virus and Japanese encephalitis, as well as bancroftian filariasis, a parasitic disease^26^.

### Viral diversity

Of the 23 eligible studies, only seven showed positive results for the detection of human pathogens, such as Japanese encephalitis virus (JEV)^27-30^, dengue type 2^26^, dengue type 3^6^, ZIKV^31^. Also, seven studies reported not detecting any human or animal pathogenic viruses in the samples. Notably, the results showed the characterization of the viral diversity present in the different species of Culicidae, the focus of each study.

Three studies described the genetic characterization of new viruses, such as Reuter *et al.* ^32^, who identified three strains of a rhabdovirus isolated from three pools of mosquitoes of the genus *Aedes* and subgenus *Ochlerotatus* sp. collected in Hungary. Similarly, the studies by Shahhosseini *et al.* ^33^ also focused on characterizing the genome and screening a new rhabdovirus, which was identified in *Ae. cantans* mosquitoes collected in Germany. The studies by Ribeiro *et al.* ^34^ identified strains of *Hubei reo-like virus 7* (HRLV 7), known as an ISV detected in one pool of *A. aegypti* and five pools of *Cx. quinquefasciatus* in Brazil.

All studies describe, in their methodological procedures, a sequence of events that encompass the choice of collection environments (peri-domestic areas, forest areas, urban or rural areas, farms, corrals, etc.), mainly those with a history of pathogen incidence, mosquito capture, as well as storage and transport, taxonomic identification, pool organization, sample processing, genomic sequencing, bioinformatics analyses, and phylogenetic analysis. Few differences were observed in the routine methodological procedures for each research laboratory.

The sample of mosquitoes that compose the pools aims to elucidate the characteristics of viromes of mosquitoes from different locations, in order to represent viral diversity and abundance during specific periods and reflect the compositions of the virome with the goal of understanding virus transmission dynamics when compared to disease outbreaks^3^.

In all studies, genomic sequencing was applied, resulting in the detection of ISVs, human pathogenic viruses, animal viruses, plant viruses, bacteriophages and a small percentage of environmental viruses, but which integrate the virome of several mosquito species^26^. Table 2 shows the various viral families.

**Table 2:** Main results of the studies included in the systematic review

	Article	Sequencing platform.	Main results of viruses detected using metagenomics
1.	Sanborn *et al*.^30^	MiSeq (Illumina)	Two pools of mosquitoes were positive for JEV. *Bunyavirales, Luteoviridae, Orthomyxoviridae, Rhabdoviridae, Totiviridae, Virgaviridae Picornaviridae, Flaviviridae* and unknown classifications.
2.	Williams *et al*.^21^	HiSeq 4000 (Illumina)	Six viral classified families and five unclassified. Only one Jogalong virus, from the *Flaviviridae* family, genus *Hepacivirus*, shares identity with viruses associated with vertebrates.
3.	Calle-Tobón *et al*.^6^	Novaseq 6000 S4 (Illumina)	Dengue fever (DENV3). Nine viral families: *Flaviviridae, Totiviridae, Reoviridae, Phenuiviridae, Iflaviridae, Bromoviridae, Metaviridae, Xinmoviridae* and *Orthomyxoviridae*. Six viruses remained unclassified.
4.	Reuter *et al*.^32^	Miseq (Illumina)	Three highly similar virus strains of a new rhabdovirus (family *Rhabdoviridae*) called Riverside virus (RISV, KU248085-KU248087) were detected and genetically characterized.
5.	Öhlund *et al*.^35^	Ion S5 XL	A large proportion of the viral readings were identified as unclassified RNA viruses. The classified viral readings belonged to the families *Iflaviridae, Nodaviridae, Orthomyxoviridae, Partitviridae, Peribunyaviridae, Phasmaviridae, Reoviridae, Rhabdoviridae, Solemoviridae* and *Tombusviridae*. De novo assembly of the viral readings produced the almost complete genomes of nine viruses in the orders *Picornavirales, Tymovirales, Bunyavirales* and *Articulavirales*, and the kingdom *Riboviria*.
6.	Kubacki *et al* ^.^2	NovaSeq (Illumina)	No human pathogenic viruses were detected. Viral sequences belonging to the families *Flaviviridae, Rhabdoviridae, Iflaviridae, Orthomyxoviridae, Dicistroviridae, Tymoviridae, Genomoviridae* and several unclassified viral taxa.
7.	He *et al* ^.^3	NovaSeq and Miseq (Illumina)	Viral readings attributed to JEV, *Culex Flavivirus* and *Quang Binh virus* belonging to the family *Flaviviridae*, genus *orthoflavivirus.* A total of 31 viral families and 116 viral species. A total of 46 species of mosquito-borne viruses belonging to 13 viral families were discovered in mosquitoes.
8.	Thannesberger *et al*.^31^	MiSeq and HiSeq (Illumina)	New variants of the ZIKV.
9.	Omuoyo *et al*.^22^	MiSeq (Illumina)	No pathogenic viruses were identified. A total of 16 viral families: *Phenuiviridaeque, Flaviviridae, Reoviridae, Baculoviridae, Closteroviridae, Iridoviridae, Orthomyxoviridae, Microviridae, Leviviridae, Siphoviridae, Picornaviridae, Iflaviviridae, Podoviridae, Peribunyaviridae, Pneumoviridae* and *Phycodnaviridae.*
10.	Sadeghi *et al*.^40^	HiSeq 2500 (Illumina)	No known human or animal pathogenic viruses were detected. The genomes of 43 RNA viruses and 16 DNA viruses were characterized and phylogenetically analyzed. The families *Alphatetraviridae, Bunyaviridae, Dicistroviridae, Flaviviridae, Iflaviridae, Luteoviridae, Mesoniviridae, Nodaviridae, Rhabdoviridae, Tombusviridae, Tymoviridae, Virgaviridae*, and the order *Picornavirales.*
11.	Ribeiro *et al*.^34^	HiSeq 2500 (Illumina)	Detection and phylogenetic characterization of Hubei reo-like virus 7 (HRLV 7) strains.
12.	Oguzie *et al*.^39^	Miseq (Illumina)	Virus families; *Bunyavirales* (*Phasi Charoen-like viruses*), *Iflaviridae* (*Tesano Aedes virus*), *Partitiviridae* (*Chaq-like viruses* and *Verdadero viruses*), *Flaviviridae* (*Cell fusion agent viruses*) and *Totiviridae* (*Aedes aegypti totivirus* (AaTV) were detected.
13.	Atoni *et al*.^26^	HiSeq 2500 (Illumina)	Dengue fever 2. A total of 30 virus families and a general group of unclassified viruses. The vertebrate virus families include *Adenoviridae, Anelloviridae, Circoviridae, Coronaviridae, Flaviviridae, Herpesviridae, Papillomaviridae, Reoviridae, Retroviridae*, and *Togaviridae*.
14.	Faizah *et al*.^28^	Miseq (Illumina)	One pool positive for JEV. A total of 27 viruses identified. Eight viruses were detected in all the pools. belonging to the families *Partitiviridae, Flaviviridae, Totiviridae* and unclassified viruses.
15.	Hameed *et al*.^29^	SBS (Illumina)*	Nine pools infected with JEV. A total of 19 viral taxonomic families with varying prevalence, including *Circoviridae, Genomoviridae, Herpesviridae, Flaviviridae, Podoviridae, Solemoviridae, Parvoviridae, Siphoviridae, Myoviridae, Nodaviridae, Luteoviridae, Retroviridae, Polydnaviridae, Microviridae, Iflaviridae, Rhabdoviridae, Totiviridae, Ackermannvirida, Peribunyaviridae*, and unclassified viruses.
16.	Sanborn *et al*.^30^	Miseq (Illumina)	A total of 20 classified species belonging to the families *Alphaflexiviridae, Alphatetraviridae, Arenaviridae, Baculoviridae, Betaflexiviridae, Birnaviridae, Dicistroviridae, Flaviviridae, Iflaviridae, Mesoniviridae, Nodaviridae, Partitiviridae, Parvoviridae, Reoviridae, Rhabdovirida, Togaviridae, Tombusviridae, Totivirida*e and *Tymoviridae*. New virus taxa.
17.	Shahhosseini *et al*.^33^	Miseq (Illumina)	Detection, characterization and screening of a new rhabdovirus.
18.	Mbigha *et al*.^36^	NextSeq500 High throughput (Illumina)	Among the 37 eukaryotic viruses identified, 26 belong to established viral families. The identified viruses belonging to families known to infect mosquitoes and insects, such as *Xinmoviridae*, *Iflaviridae*, and *Phasmaviridae*, likely represent true mosquito-infecting viruses. Viruses belonging to families containing known arboviruses, such as *Flaviviridae* (*Menghai flavivirus*) and *Peribunyaviridae* (*Duke bunyavirus*), suggesting potential transmission risks to arthropods and vertebrates, including humans.
19.	Gómez *et al*.^38^	Oxford Nanopore Technologies (ONT)	The families *Phenuiviridae, Flaviviridae, Partitiviridae, Rhabdoviridae, Picornaviridae, Iflaviridae, Peribunyaviridae*, and *Togaviridae*.
20.	Guimarães *et al*.^43^	Miseq (Illumina)	The mosquito virome was abundant in the families *Picornavirales, Iflaviridae, Nodamuvirales, Nodaviridae, Sobelivirales,* and *Solemoviridae*.
21.	Li *et al*.^45^	Miseq (Illumina)	The identified viruses were from 12 established viral families, including *Dicistroviridae, Flaviviridae, Hantaviridae, Iridoviridae, Narnaviridae, Partitiviridae, Peribunyaviridae, Phasmaviridae, Rhabdoviridae, Sedoreoviridae, Solemoviridae, Totiviridae*, and unclassified *Bunyavirales*, unclassified *Riboviria*, or unclassified viruses.
22.	Guimarães *et al*.^46^	Miseq (Illumina)	Characterization of the genome of a new species of *iflavirus*. provisionally named *Iflavirus desanae*.
23.	De Coninck *et al*.^47^	Nextseq 550 (Illumina)	The observed viral species belonged to 23 different viral families. The viral families were present in multiple locations, for example, *Chrysoviridae, Nodaviridae*, and *Orthomyxoviridae.*

*SBS (Illumina): sequencing by synthesis (SBS), is a next-generation sequencing (NGS) technology, in which Illumina instruments and reagents use a proprietary method that detects single bases as they are incorporated into growing DNA strands with massively parallel capabilities. Currently available on the MiSeq i100 platforms, the NextSeq 1000 and NextSeq 2000 Systems, and the NovaSeq X Series.

The main sequencers used in the studies were NovaSeq, Miseq and HiSeq (Illumina), with only one study using Ion Torrent S5 XL (Thermo Fisher Scientific, Waltham, MA, USA)^35^.

As for virus families, viral species of medical and veterinary importance identified as part of mosquito viromes were described, belonging to 53 virus families, as well as unclassified virus sequences. After analyzing the 23 studies, vertebrate viruses known to infect mammals naturally were identified, belonging to the following families: *Adenoviridae, Anelloviridae, Circoviridae, Coronaviridae, Flaviviridae, Herpesviridae, Papillomaviridae, Reoviridae, Retroviridae, Togaviridae*, and *Rhabdoviridae* ^26,30^.

The *Flaviviridae* family was detected in 19 studies, represented by flaviviruses that infect vertebrates and some insect viruses^26^. It was observed that the detection of sequences belonging to the *Flaviviridae* family is important due to the high number of sequences identified and their phylogenetic proximity to known pathogens, such as the St. Louis encephalitis virus (SLEV; *Flaviviridae*: *Orthoflavivirus*), the West Nile virus (WNV; *Flaviviridae*: Orthoflavivirus), the Zika virus (ZIKV; *Flaviviridae: Orthoflavivirus*), and the dengue virus (DENV; *Flaviviridae*: *Orthoflavivirus*).

The studies highlight the importance of research into ISVs, which are known to be non-pathogenic, and infect and replicate exclusively in insects^36^. Although these insect-specific viruses do not seem to replicate in vertebrates, some of them are phylogenetically related to known pathogenic viruses. It is speculated that they may evolve into new pathogenic viruses in vertebrates^30,37,38^.

Thus, the studies by Calle-Tobón *et al*.^6^ detected ISVs in *Aedes aegypti* samples, which belonged to the *Totiviridae* family (*Aedes aegypti toti-like virus* and *Australian Anopheles totivirus*), and an ISV from the *Iflaviridae* family (*iflavirus*). The study by Oguzie *et al*.^39^ detected the *Tesano Aedes virus* (TeAV), a member of the *Iflaviridae* family, in mosquito pools. Similarly, Kubacki *et al*.^2^ detected members of the *Dicistroviridae* family.

The abundance of viral readings varied greatly according to the virus classification and mosquito species. Species from viral families known to be associated with plants were detected, and among them, members of the *Virgaviridae*, *Sobemoviridae* families^30^, the *Tombusviridae* family^35^, the *Tymovirdae* family^2^ and the *Luteoviridae* family^40^.

Viral sequences belonging to the *Mimiviridae* and *Totiviridae* families, known to infect protozoa, were also detected^26^. Overall, unclassified viruses represented a considerable number of viral sequences, indicating the need to study them to understand their genetic, ecological and evolutionary diversity and their ability to cause future outbreaks^29^.

### Quality of evidence

Information regarding the quality of evidence/risk of bias was based on the Systematic Review Center for Laboratory Animal Experimentation (SYRCLE) tool for animal studies^20^ and adapted according to its guidelines, which recommend that researchers and collaborators who are going to assess the risk of bias of the included studies discuss it in advance and adapt it to the specific needs of their review^20^.

All 23 studies included in this systematic review were primary, experimental, cross-sectional and prospective. Although this review had limitations in terms of assessing methodological quality, all studies were classified as having a low risk of bias.

The risk of selection bias refers to studies involving animals and relates to the methodology used to select and divide specific groups of animals in order to avoid distortions. Selection bias was assessed across three domains: sequence generation, baseline characteristics, and allocation concealment. The first domain, sequence generation, relates to whether the description was sufficient to allow for an assessment and whether it enabled the formation of comparable groups^20^. In this regard, all studies identified mosquitoes by species and allocated them into groups (pools), as shown in Table 1.

The second domain concerns baseline characteristics, described as all possible prognostic factors or characteristics of the animals, which, in other cases, can be compared to assess whether the intervention and control groups were similar at the start of the experiment^20^. In all the articles included in this study, the groups were standardized regarding specimens number, stored under refrigeration for the preservation of viral DNA or RNA, and subsequently processed for metagenomic sequencing. The sequence generation and baseline characteristics, which were related to selection bias, of the domains were considered to have a low risk of bias (YES) in 100% of the studies.

The third domain concerns allocation concealment, referring to the method used to conceal the allocation sequence. It is necessary to provide sufficient details to determine whether intervention allocations could have been predicted before or during enrollment^20^. This domain showed a high risk of bias (NO) in 100% of the studies, as there was no concealment in groups allocation. The lack of concealment can be attributed to the need to identify the mosquitoes down to the species level as this information was essential to understand the diversity and ecology of the viruses that would be found after genomic sequencing.

Performance bias in animal studies is related to the conduct of the study, in which the method of administering substances or handling animals may influence the results, leading to incorrect or distorted conclusions. Performance bias is presented across two domains: random housing and blinding. The random housing domain assesses the measures used to randomly house the animals, while the blinding domain refers to the measures used to prevent the researchers from knowing which intervention each animal received^20^.

In all the articles, the groups were distributed by species and stored under refrigeration, without random housing. Regarding blinding, it was important for the researcher to know the mosquito species, therefore no intervention was performed. Therefore, a high risk of bias (NO) was observed for both domains in 100% of the studies.

Detection bias, on the other hand, is related to the possibility of identifying an effect of the intervention, mainly due to factors that influence how the outcomes are measured or observed. It is represented by two domains: random outcome assessment and blinding. Random outcome assessment refers to whether the animals were described as being randomly selected for outcome evaluation and which methods were used for their selection. In this study, the assessment was carried out randomly based on the detection of viruses in the samples.

In the blinding domain, it is proposed to identify all measures used to blind the outcome raters, preventing them from knowing which intervention each animal received^20^. It is understood that blinding was achieved by employing metagenomic techniques to detect all viral sequences present in the samples. Both domains showed a low risk of bias (YES) in 100% of the studies.

Attrition bias occurs when there is a systematic loss of animals between the study groups that are a part of the research over time, leading to distorted results. It is represented by only one domain: incomplete outcome data. This domain is related to losses and exclusions from the analysis that directly impact the primary outcome^20^. None of the articles mentioned the exclusion of mosquito groups in the outcome evaluation, and 100% of the studies were classified as having an uncertain risk of bias in this domain.

For reporting bias, it is understood that study results may be distorted or omitted due to the influence of factors such as researchers’ interests, the significance of the results, or a preference for reporting findings that support certain conclusions. Only one domain is presented: selective outcome reporting, which aims to determine how the examination was conducted and how the results were identified^20^. In 100% of the studies, the presence of the virus was detected, representing a low risk of bias (YES).

Other sources of bias, represented by the domain other sources of bias, regarding important concerns about biases not addressed by other domains of the SYRCLE tool are left to the researchers’ judgment^20^. None of the articles presented other sources of bias, thus showing a low risk of bias. Therefore, presenting a low risk of bias (YES) in 100% of the studies.

## DISCUSSION

To search for evidence of the effectiveness of the viral metagenomics technique in detecting arboviruses in mosquitoes, this systematic review identified 23 articles that obtained promising results in detecting arboviruses in mosquitoes. Furthermore the search strategy led to the knowledge of the diversity of virus species in more than 53 viral families.

The eligible studies that make up this systematic review used the viral metagenomics technique as an important tool from the perspective of pathogen surveillance and control^41,42^, proving to be efficient in identifying arboviruses due to the sensitivity in detecting all viral genomes that coexist in a given sample^14^, in addition to playing a relevant role in understanding viral diversity and supporting studies that clarify the ecological function that viruses perform in the environment^3,30,43^.

Arboviruses (Arthropod-borne viruses) are viruses transmitted in nature by hematophagous arthropods, with emphasis on dipterans of the Culicidae family, which are often involved in complex cycles involving vertebrates, such as mammals or birds. Both humans and animals may be infected and present diseases ranging from subclinical cases to fevers, encephalitis, and hemorrhagic diseases with a significant proportion of fatalities^44^.

RNA viruses are directly related to the cause of new diseases, as the mutation rate is predominantly high, enabling adaptation to new hosts^2^, increasing the transmissibility of viruses by several species of mosquitoes, directly contributing to their geographic distribution^3^. In the last decade, deep sequencing technologies have further enabled the discovery of newly identified RNA viruses associated with arthropods, including those discovered in mosquitoes^33,45^.

Thus, the richness and abundance of Culicidae found in a given area—especially areas with a history of arbovirus outbreaks and environments conducive to mosquito breeding—constitute an important reservoir for many different viruses, contributing to their spread and evolution^3,46^. Mirroring the virome of Culicidae populations via metagenomic analysis aims to explore the spatial and temporal dynamics of virus diversity in mosquitoes, to understand virus–virus interactions and identify new strategies for preventing arbovirus diseases^34^.

Therefore, the mosquito virome arouses interest due to the possibility of detecting new viruses, which enables a critical look at the ecology of emerging and reemerging pathogens that circulate in vector populations^21,47^. In particular, the vectors of human viruses are relevant for identifying potential areas of disease outbreaks and for understanding the dynamism of mosquitoes in the genetic evolution of viruses, offering observable ecological correlates and attracting permanent surveillance attention via the viral metagenomics of mosquitoes^2,30,45^.

The worldwide distribution and population density of Culicidae favor viral transmission and increase the risk of viral diseases. Among the various species shown in Table 1, *Ae. albopictus* and *Ae. aegypti* are vectors of clinically important viruses, such as ZIKV, DENV, CHIKV and WNV^2^.

In Medellin, Colombia, Calle-Tobón *et al*.^6^ detected 21 types of viruses related to ISVs, arboviruses and plant viruses belonging to nine different viral families. By applying conducting metagenomics assays in the species *Ae. aegypti* and *Ae. Albopictus* the *Flaviviridae, Totiviridae, Reoviridae, Phenuiviridae, Iflaviridae, Bromoviridae, Metaviridae, Xinmoviridae* and *Orthomyxoviridae* families were identified; however, six viruses remained unclassified. Only one *Ae. aegypti* sample was positive for DENV3.

According to Atoni *et al*.^26^, in their investigation carried out in Kenya and southwest China, 240 pools were grouped predominantly of *Culex quinquefasciatus* and *Culex tritaeniorhynchus* species. After genome sequencing, followed by bioinformatics analysis, a total of 30 virus families and a general group of unclassified viruses were detected. The vertebrate viruses known to naturally infect mammals and replicate in mammalian cell lines are highlighted, including *Adenoviridae, Anelloviridae, Circoviridae, Coronaviridae, Flaviviridae, Herpesviridae, Papillomaviridae, Reoviridae, Retroviridae, Togaviridae* and *Rhabdoviridae*. The dengue fever 2 virus was detected in a sample in China and later confirmed by polymerase chain reaction (PCR).

Thannesberger *et al*.^31^ focused on mosquito collection in Barbados during the peak of the 2016 ZIKV epidemic, using viral metagenomics in *Ae. aegypt*i mosquitoes, resulting in the detection of two distinct ZIKV genotypes.

Among other eligible studies, Faizah *et al*.^28^ collected mosquitoes in Japan to isolate the JEV, processing samples from *Cx. tritaeniorhynchus, Cx. pseudovishnui, Cx. vishnui* and *Cx. inatomii* mosquitoes. The metagenomic analyses comprised double-stranded RNA (dsRNA) viruses belonging to the *Totiviridae, Partitiviridae* and *Chrysoviridae* families; positive-sense (+) single-stranded RNA viruses (ssRNA) belonging to the *Flaviviridae* and *Iflaviridae* families and unclassified groups; and negative-sense (−) ssRNA viruses from the *Xinmoviridae* and *Rhabdoviridae* families and unclassified groups.

Other studies that showed positive results for JEV were conducted in the Yunnan–Myanmar border region of China, where *Cx. Tritaeniorhyncus, Ae. aegypti, An. sinensis* and *Ar. subalbatus* species were subjected to next-generation sequencing (NGS)^29^. In addition, the species *Culex pipiens, Culex tritaeniorhynchus, Anopheles sinensis,* and *Aedes* sp were collected in the Shaanxi-Gansu-Ningxia region^3^. NGS was conducted on 260 pools of *Cx. tritaeniorhynchus* and *Cx. bitaeniorhynchus* mosquitoes collected in Camp Humphreys, Republic of Korea^30^.

In this sense, technological advances in NGS have provided an expansion of understanding of the richness, abundance, viral evolution and dynamics of pathogens present in mosquitoes^3,6,21^. This technology makes it possible to identify any viruses in a given sample without laboratory culture and viral isolation. Therefore, this tool holds importance in the discovery and surveillance of viruses of medical and veterinary importance^3,29,30,48^.

With NGS, the nucleic acids present in a given sample are sequenced and analyzed using bioinformatics to identify viral sequences according to reference genomic databases. Thus, sequencing platforms are sensitive in detecting arboviruses, providing information on genetic evolution or sequence similarity in relation to viral marker genes^31^.

When discussing the application of metagenomics in the detection and characterization of new viruses, the study by Shahhosseini *et al.* ^33^ using a pool composed of 25 *Aedes cantans* females, a mosquito species known to be involved in the transmission of several arboviruses in Europe, were captured in the city of Hamburg, Germany, and were positive for rhabdovirus after being subjected to metagenomic analysis. The virus was genetically related to recently discovered mosquito-associated rhabdoviruses. Reuter *et al.* ^32^ also detected three pools of mosquitoes positive for a new rhabdovirus that contained only *Ochlerotatus* sp. mosquitoes collected in Hungary, which were detected and genetically characterized.

Among the results presented in Table 2, the viruses belonging to the genera *Alphavirus* of the *Togaviridae* family and Ortho*flavivirus* of Flaviviridae family stand out, as well as other arboviruses of clinical importance that belong to the families *Bunyaviridae, Reoviridae* and *Rhabdoviridae* ^9^.

Faced with the diversity of constantly mutating virus populations, metagenomics enables the comparison of virulence characteristics with available genomic databases, determining susceptibility to diseases and enabling their tracking and control; This technique also make it possible to predict and prevent future threats to public health^3^.

In addition to arboviruses, ISVs are also addressed in the studies included in this systematic review. With the improvement of sequencing platforms, the investigation and discovery of insect-specific viruses has been significantly expanded via metagenomics. Mosquitoes have been the main focus of ISV screening^49^.

ISVs are restricted to arthropods and cannot replicate in vertebrate cells or tissues^22^. The prevalence of ISVs in wild mosquito populations is underscored by their ability to suppress, increase or have no effect on the replication of medically important arboviruses, potentially affecting vector competence, which contributes to their use as biocontrol agents^48^.

Although the mechanism by which ISVs modulate arboviruses is unclear, no ISV is known to prevent transmission of all common arboviruses of veterinary and human importance^22,40,41^. In this sense, many ISVs are shown to be genetically related to the phylogeny of pathogenic viruses. With this genetic similarity, it is possible to clarify the potential for interference in the transmission of pathogens or the dynamics of replication in the vector, in which similar viruses can block each other due to competition^45,48^.

Thus, we envision studies that could lead to advances in the understanding of ISVs, especially related to their effects on arbovirus infection and transmission, and in the use of mechanisms of biological vector control, as well as fostering the emergence of new vaccine platforms^40,41^.

Among the diversity of viruses detected in the mosquito virome, the studies highlight the abundant detection of unclassified viruses^50^, stressed by recent advances in random amplification technologies. Metagenomic surveillance has enabled the expansion in the number of new, and often unclassified, viruses^33^.

NGS advances have enabled the molecular and taxonomic identification of viruses, assigned by the International Committee on Taxonomy of Viruses (ICTV). However, the results of the comparison of the viral content of the contigs are generally categorized as unclassified viruses, due to the need for studies that show coherent information regarding their ecology and morphological composition^26^.

There is little information available on unclassified viruses and their evolution in mosquitoes. The discovery and characterization of these viruses highlights the need for constant vigilance in preventing future outbreaks, due to the potential for mutation, which contributes to the emergence of new diseases in vertebrates^29,46^.

One of the main challenges highlighted in metagenomic studies is the limited sequencing information for viral families and genera available in databases^6^. Thus, the lack of reference sequences for most mosquito species is also a factor against the computational removal of RNA readings^42^.

Another problem raised in the studies refers to the metagenomic analysis of entire mosquitoes, due to the amount of viruses that can be included in the analysis, such as plant viruses belonging to their diet and viruses from parasitic or commensal organisms that reside inside or under the mosquito^35^.

## CONCLUSION

This systematic review demonstrated the effectiveness of viral metagenomics in the detection of arboviruses—as evidenced by the 23 eligible studies included—both in the identification of pathogens and in the characterization of viral diversity, with the aim of understanding ecological and epidemiological aspects of these viruses. Metagenomics has emerged as an essential tool for expanding the taxonomic identification of viruses, based on studies focused on understanding viral genomes in recent years.

Metagenomics has simplified the detection of viruses in mosquito samples, enabling constant epidemiological surveillance, predicting and preventing outbreaks due to its sensitivity in detecting viral sequences without the need for a large sample and isolation by culture. On the other hand, the high financial costs of acquiring and maintaining NGS technology restrict its use to a few research laboratories. Despite the limited studies on a global scale, there is a need to deepen the understanding of the mosquito virome in order to prevent future epidemics that could impact social, economic, and health needs in the context of globalization.

## References

[1] Fauci AS, Morens DM (2016). Zika virus in the Americas: yet another arbovirus threat. N Engl J Med.

[2] Kubacki J, Flacio E, Qi W, Guidi V, Tonolla M, Fraefel C (2020). Viral metagenomic analysis of Aedes albopictus mosquitos from Southern Switzerland. Viruses.

[3] He X, Yin Q, Zhou L, Meng L, Hu W, Li F (2021). Metagenomic sequencing reveals viral abundance and diversity in mosquitoes from the Shaanxi-Gansu-Ningxia region, China. PLoS Negl Trop Dis.

[4] Lopes N, Nozawa C, Linhares RE (2014). Características gerais e epidemiologia dos arbovírus emergentes no Brasil. Rev Pan-Amaz Saude.

[5] Terzian AC, Mondini A, Bronzoni RV, Drumond BP, Ferro BP, Cabrera EM (2011). Detection of Saint Louis encephalitis virus in dengue-suspected cases during a dengue 3 outbreak. Vector Borne Zoonotic Dis.

[6] Calle-Tobón A, Pérez-Pérez J, Forero-Pineda N, Chávez OT, Rojas-Montoya W, Rúa-Uribe G (2022). Local-scale virome depiction in Medellín, Colombia, supports significant differences between Aedes aegypti and Aedes albopictus. PloS One.

[7] Ribeiro AC, Carvalho CA, Casseb SM, Rodrigues SG, Vasconcelos PF, Carvalho VL (2018). Infection profiles of Mayaro virus and Chikungunya virus in mammalian and mosquito cell lineages. Rev Pan-Amaz Saude.

[8] Alencar J, Pacheco JB, Correa FF, Silva JS, Guimarães AE (2011). New report on the bionomics of Coquillettidia venezuelensis in temporary breeding sites (Diptera: Culicidae). Rev Soc Bras Med Trop.

[9] Donalisio MR, Freitas AR, Zuben AP (2017). Arboviruses emerging in Brazil: challenges for clinic and implications for public health. Rev Saude Publica.

[10] Saivish MV, Costa VG, Menezes GL, Silva RA, Silva GC, Moreli ML (2021). Rocio virus: an updated view on an elusive flavivirus. Viruses.

[11] Silva FA, Ferreira MS, Araújo PA, Casseb SM, Silva SP, Nunes JP (2022). Serological and molecular evidence of the circulation of the Venezuelan equine encephalitis virus subtype IIIA in humans, wild vertebrates and mosquitos in the Brazilian Amazon. Viruses.

[12] Cilli A, Luchs A, Leal E, Gill D, Milagres FA, Komninakis SV (2019). Human sapovirus GI.2 and GI.3 from children with acute gastroenteritis in northern Brazil. Mem Inst Oswaldo Cruz.

[13] Dávila-Ramos S, Castelán-Sánchez HG, Martínez-Ávila L, Sánchez-Carbente MD, Peralta R, Hernández-Mendoza A (2019). A review on viral metagenomics in extreme environments. Front Microbiol.

[14] Carroll D, Daszak P, Wolfe ND, Gao GF, Morel CM, Morzaria S (2018). The Global Virome Project. Science.

[15] Page MJ, McKenzie JE, Bossuyt PM, Boutron I, Hoffmann TC, Mulrow CD (2022). A declaração PRISMA 2020: diretriz atualizada para relatar revisões sistemáticas. Rev Panam Salud Publica.

[16] Felipe LR, Barbosa KS, Virtuoso JS (2022). Sintomatologia depressiva e mortalidade em idosos da América Latina: uma revisão sistemática com metanálise. Rev Panam Salud Publica.

[17] Cassiani SH, Fernandes MN, Reveiz L, Freire JR, Silva FA (2020). Combinação de tarefas do enfermeiro e de outros profissionais na atenção primária em saúde: uma revisão sistemática. Rev Panam Salud Publica.

[18] Cardozo NO, Crisp AH, Pinheiro FA, Trude AC, Araneda-Flores J, Oliveira MR (2022). Ambiente alimentar e excesso de peso em escolares: uma revisão sistemática sul-americana. Rev Panam Salud Publica.

[19] Hosseini MS, Jahanshahlou F, Akbarzadeh MA, Zarei M, Vaez-Gharamaleki Y (2024). Formulating research questions for evidence-based studies. J Med Surg Public Health.

[20] Hooijmans CR, Rovers MM, de Vries RB, Leenaars M, Ritskes-Hoitinga M, Langendam MW (2014). SYRCLE's risk of bias tool for animal studies. BMC Med Res Methodol.

[21] Williams SH, Levy A, Yates RA, Somaweera N, Neville PJ, Nicholson J (2020). The diversity and distribution of viruses associated with Culex annulirostris mosquitoes from the Kimberley Region of Western Australia. Viruses.

[22] Omuoyo DO, Nyamwaya DK, Kamau E, Nyagwange JN, Karanja HK, Gitonga JN (2023). Metagenomic analysis of coastal Kenya female Aedes aegypti mosquito RNA metaviromes reveal presence of diverse insect specific viruses. Wellcome Open Res.

[23] Silva CM, Sérvio HS, Ramos RA, Faustino MA, Alves LC, Carvalho GA (2014). Occurrence of immature forms of culicids (Insecta: Diptera) in the northeastern region of Brazil. Rev Bras Parasitol Vet.

[24] Silva AF, Machado LC, Paula MB, Vieira CJ, Bronzoni RV, Santos MA (2020). Culicidae evolutionary history focusing on the Culicinae subfamily based on mitochondrial phylogenomics. Sci Rep.

[25] Reinhold J, Lazzari C, Lahondère C (2018). Effects of the environmental temperature on Aedes aegypti and Aedes albopictus mosquitoes: a review. Insects.

[26] Atoni E, Wang Y, Karungu S, Waruhiu C, Zohaib A, Obanda V (2018). Metagenomic virome analysis of Culex mosquitoes from Kenya and China. Viruses.

[27] He X, Yin Q, Zhou L, Meng L, Hu W, Li F (2021). Metagenomic sequencing reveals viral abundance and diversity in mosquitoes from the Shaanxi-Gansu-Ningxia region, China. PLoS Negl Trop Dis.

[28] Faizah AN, Kobayashi D, Isawa H, Amoa-Bosompem M, Murota K, Higa Y (2020). Deciphering the virome of Culex vishnui subgroup mosquitoes, the major vectors of Japanese encephalitis, in Japan. Viruses.

[29] Hameed M, Wahaab A, Shan T, Wang X, Khan S, Di D (2021). A metagenomic analysis of mosquito virome collected from different animal farms at Yunnan-Myanmar border of China. Front Microbiol.

[30] Sanborn MA, Wuertz KM, Kim HC, Yang Y, Li T, Pollett SD (2021). Identification of Japanese encephalitis virus genotype V and other mosquito-borne viruses in Camp Humphreys, Republic of Korea, using metagenomic analysis. bioRxiv.

[31] Thannesberger J, Rascovan N, Eisenmann A, Klymiuk I, Zittra C, Fuehrer HP (2021). Viral metagenomics reveals the presence of novel Zika virus variants in Aedes mosquitoes from Barbados. Parasit Vectors.

[32] Reuter G, Boros A, Pál J, Kapusinszky B, Delwart E, Pankovics P (2016). Detection and genome analysis of a novel (dima)rhabdovirus (Riverside virus) from Ochlerotatus sp. mosquitoes in Central Europe. Infect Genet Evol.

[33] Shahhosseini N, Lühken R, Jöst H, Jansen S, Börstler J, Rieger T (2017). Detection and characterization of a novel rhabdovirus in Aedes cantans mosquitoes and evidence for a mosquito-associated new genus in the family Rhabdoviridae. Infect Genet Evol.

[34] Ribeiro GO, Monteiro FJ, Rego MO, Ribeiro ES, Castro DF, Caseiro MM (2019). Detection of RNA-dependent RNA polymerase of Hubei Reo-like virus 7 by next-generation sequencing in Aedes aegypti and Culex quinquefasciatus mosquitoes from Brazil. Viruses.

[35] Öhlund P, Hayer J, Lundén H, Hesson JC, Blomström AL (2019). Viromics reveal a number of novel RNA viruses in Swedish mosquitoes. Viruses.

[36] Mbigha Donfack KC, De Coninck L, Ghogomu SM, Matthijnssens J (2024). Aedes mosquito virome in Southwestern Cameroon: lack of core virome, but a very rich and diverse virome in Ae. africanus compared to other Aedes species. Viruses.

[37] Bolling B, Weaver S, Tesh R, Vasilakis N (2015). Insect-specific virus discovery: significance for the Arbovirus community. Viruses.

[38] Gómez M, Martínez D, Páez-Triana L, Luna N, Ramírez A, Medina J (2024). Influence of dengue virus serotypes on the abundance of Aedes aegypti insect-specific viruses (ISVs). J Virol.

[39] Oguzie JU, Nwangwu UC, Oluniyi PE, Olumade TJ, George UE, Kazeem A (2022). Metagenomic sequencing characterizes a wide diversity of viruses in field mosquito samples in Nigeria. Sci Rep.

[40] Sadeghi M, Altan E, Deng X, Barker CM, Fang Y, Coffey LL (2018). Virome of > 12 thousand Culex mosquitoes from throughout California. Virology.

[41] Shi C, Beller L, Deboutte W, Yinda KC, Delang L, Vega-Rúa A (2019). Stable distinct core eukaryotic viromes in different mosquito species from Guadeloupe, using single mosquito viral metagenomics. Microbiome.

[42] Koh C, Frangeul L, Blanc H, Ngoagouni C, Boyer S, Dussart P (2022). Decoding rRNA sequences for improved metagenomics of sylvatic mosquito species. bioRxiv.

[43] Guimarães LO, Ribeiro GO, Couto R, Ramos ES, Morais VS, Telles-de-Deus J (2025). Exploring mosquito virome dynamics within São Paulo Zoo: insights into mosquito-virus-environment interactions. Front Cell Infect Microbiol.

[44] Liang G, Gao X, Gould EA (2015). Factors responsible for the emergence of arboviruses; strategies, challenges and limitations for their control. Emerg Microbes Infect.

[45] Li T, Alam MS, Yang Y, Al-Amin HM, Rahman M, Islam F (2025). Metagenome analysis of viruses associated with Anopheles mosquitoes from Ramu Upazila, Cox's Bazar District, Bangladesh. PeerJ.

[46] Guimarães LO, Bahia SL, Ribeiro GO, Ramos ES, Villanova F, Morais VS (2024). New iflavirus species characterized from mosquitoes captured in the Sao Paulo zoological facilities. Microorganisms.

[47] Coninck L, Soto A, Wang L, Wolf K, Smitz N, Deblauwe I (2024). Lack of abundant core virome in Culex mosquitoes from a temperate climate region despite a mosquito species-specific virome. mSystems.

[48] Ramos-Nino ME, Fitzpatrick DM, Tighe S, Eckstrom KM, Hattaway LM, Hsueh AN (2020). High prevalence of Phasi Charoen-like virus from wild-caught Aedes aegypti in Grenada, W.I. as revealed by metagenomic analysis. PLoS One.

[49] Öncü C, Brinkmann A, Günay F, Kar S, Öter K, Sarikaya Y (2018). West Nile virus, Anopheles flavivirus, a novel flavivirus as well as Merida-like rhabdovirus Turkey in field-collected mosquitoes from Thrace and Anatolia. Infect Genet Evol.

[50] Sanborn MA, Klein TA, Kim HC, Fung CK, Figueroa KL, Yang Y (2019). Metagenomic analysis reveals three novel and prevalent mosquito viruses from a single pool of Aedes vexans nipponii collected in the Republic of Korea. Viruses.

